# Discovery of a Novel Inner Membrane-Associated Bacterial Structure Related to the Flagellar Type III Secretion System

**DOI:** 10.1128/jb.00144-22

**Published:** 2022-07-18

**Authors:** Mohammed Kaplan, Catherine M. Oikonomou, Cecily R. Wood, Georges Chreifi, Debnath Ghosal, Megan J. Dobro, Qing Yao, Ritesh Ranjan Pal, Amit K. Baidya, Yuxi Liu, Stefano Maggi, Alasdair W. McDowall, Sigal Ben-Yehuda, Ilan Rosenshine, Ariane Briegel, Morgan Beeby, Yi-Wei Chang, Carrie L. Shaffer, Grant J. Jensen

**Affiliations:** a Division of Biology and Biological Engineering, California Institute of Technology, Pasadena, California, USA; b Department of Veterinary Sciences, University of Kentucky College of Agriculture, Lexington, Kentucky, USA; c Division of Medicine, Dentistry and Health Sciences, University of Melbourne, Parkville, Victoria, Australia; d Hampshire College, Amherst, Massachusetts, USA; e Department of Microbiology and Molecular Genetics, Institute of Medical Research Israel-Canada, Faculty of Medicine, The Hebrew University of Jerusalem, Jerusalem, Israel; f School of Biological Sciences, Indian Association for the Cultivation of Science, Kolkata, West Bengal, India; g Molecular Biophysics Unit, Indian Institute of Science, Bangalore, Karnataka, India; h Leiden University, Institute of Biology, Leiden, The Netherlands; i Department of Life Sciences, Imperial College London, South Kensington Campus, London, United Kingdom; j Department of Biochemistry and Biophysics, Perelman School of Medicine, University of Pennsylvania, Philadelphia, Pennsylvania, USA; k Department of Microbiology, Immunology, and Molecular Genetics, University of Kentucky College of Medicine, Lexington, Kentucky, USA; l Department of Pharmaceutical Sciences, University of Kentucky College of Pharmacy, Lexington, Kentucky, USA; m Department of Chemistry and Biochemistry, Brigham Young University, Provo, Utah, USA; NCBI, NLM, National Institutes of Health

**Keywords:** bacteria, cryo-ET, flagella, secretion systems

## Abstract

The bacterial flagellar type III secretion system (fT3SS) is a suite of membrane-embedded and cytoplasmic proteins responsible for building the flagellar motility machinery. Homologous nonflagellar (NF-T3SS) proteins form the injectisome machinery that bacteria use to deliver effector proteins into eukaryotic cells, and other family members were recently reported to be involved in the formation of membrane nanotubes. Here, we describe a novel, evolutionarily widespread, hat-shaped structure embedded in the inner membranes of bacteria, of yet-unidentified function, that is present in species containing fT3SS. Mutant analysis suggests a relationship between this novel structure and the fT3SS, but not the NF-T3SS. While the function of this novel structure remains unknown, we hypothesize that either some of the fT3SS proteins assemble within the hat-like structure, perhaps including the fT3SS core complex, or that fT3SS components regulate other proteins that form part of this novel structure.

**IMPORTANCE** The type III secretion system (T3SS) is a fascinating suite of proteins involved in building diverse macromolecular systems, including the bacterial flagellar motility machine, the injectisome machinery that bacteria use to inject effector proteins into host cells, and probably membrane nanotubes which connect bacterial cells. Here, we accidentally discovered a novel inner membrane-associated complex related to the flagellar T3SS. Examining our lab database, which is comprised of more than 40,000 cryo-tomograms of dozens of species, we discovered that this novel structure is both ubiquitous and ancient, being present in highly divergent classes of bacteria. Discovering a novel, widespread structure related to what are among the best-studied molecular machines in bacteria will open new venues for research aiming at understanding the function and evolution of T3SS proteins.

## INTRODUCTION

**FIG 1:**
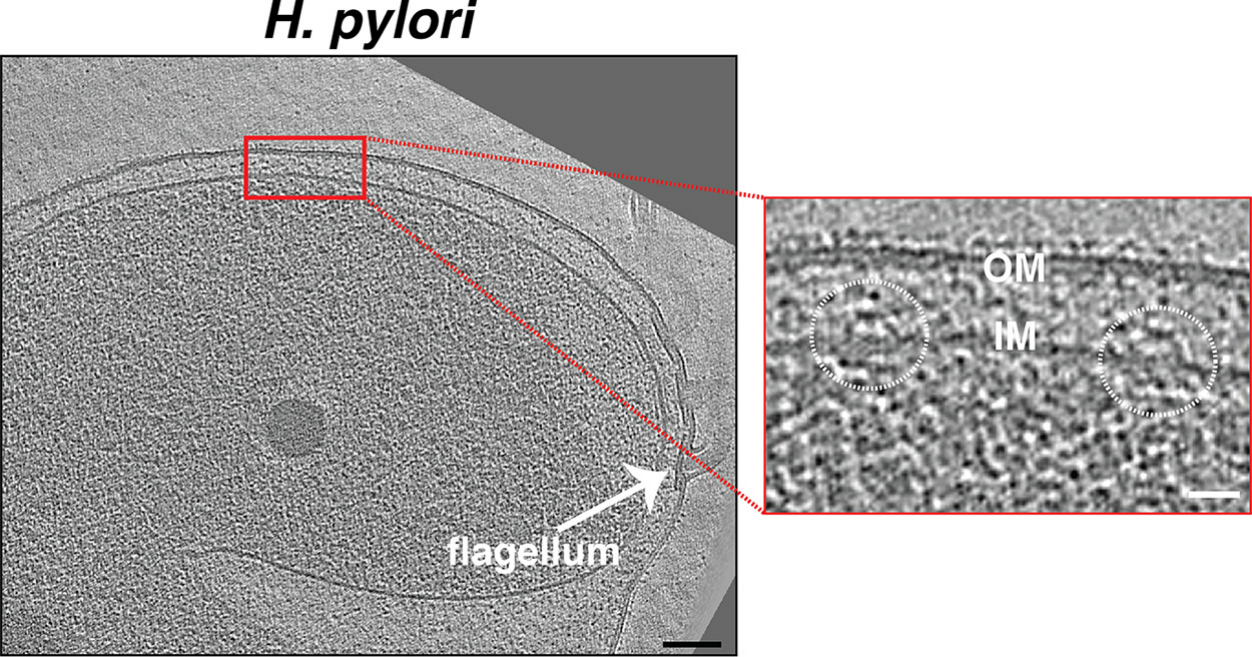
Identification of a novel hat-like structure in H. pylori. A slice through an electron cryo-tomogram of an H. pylori cell, showing two hat-like structures (white circles in the enlargement). Scale bars represent 100 nm in the main panel and 25 nm in the enlargement.

**FIG 2:**
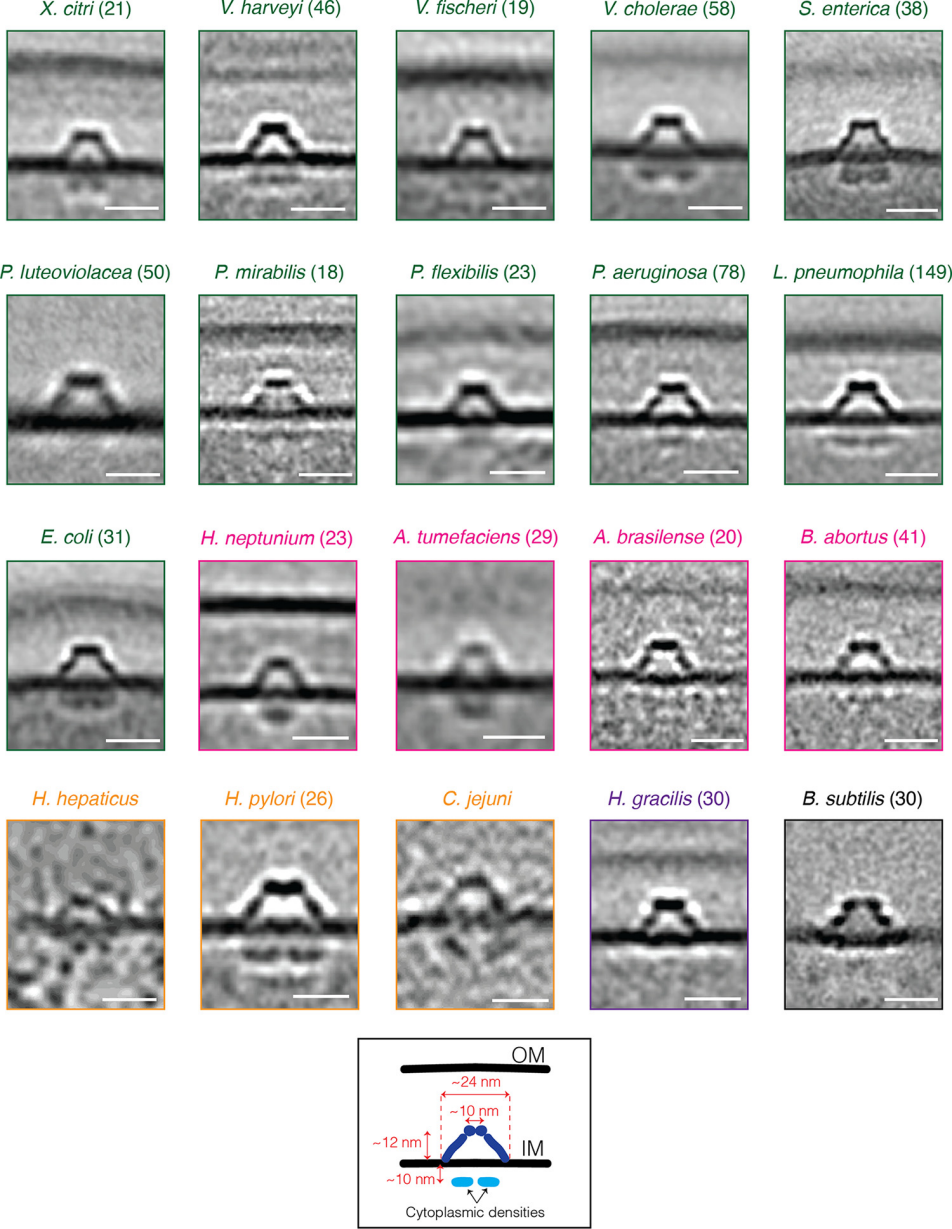
The hat-like complex is a widespread bacterial structure. A gallery of the hat-like structure in various bacterial species (Xanthomonas citri, Vibrio harveyi, V. fischeri, V. cholerae, Salmonella enterica, Pseudoalteromonas luteoviolacea, Proteus mirabilis, Pseudomonas flexibilis, P. aeruginosa, Legionella pneumophila, Escherichia coli, Hyphomonas neptunium, Agrobacterium tumefaciens, Azospirillum brasilense, Brucella abortus, Helicobacter hepaticus, H. pylori, Campylobacter jejuni, Hylemonella gracilis, and Bacillus subtilis). Subtomogram averages are shown for all species except C. jejuni and H. hepaticus, for which insufficient data were available for averaging. For these two species, single tomographic slices are shown. Numbers in parentheses indicate how many particles were used to produce the subtomogram average. Color coding indicates taxonomic class: green, Gammaproteobacteria; pink, Alphaproteobacteria; light orange, Epsilonproteobacteria; purple, Betaproteobacteria; and black, Bacilli. Scale bars represent 20 nm.

Cryogenic electron tomography (cryo-ET) images intact cells in 3D in a native, frozen-hydrated state, occasionally revealing unexpected structures (1–4). Examining a database of tens of thousands of electron cryo-tomograms of phylogenetically diverse bacterial species that our lab has imaged over the past 15 years (5, 6), we found an inner membrane (IM)-associated, hat-shaped structure in diverse Gram-negative and Gram-positive bacteria (Fig. 1 and S1). In many cases, we observed multiple such structures, up to 10, distributed around the cell (see Movie S1 for an example from an Escherichia coli cell that was partially lysed, enhancing visibility of periplasmic structures). Subtomogram averages of the structure from different species revealed conserved characteristics: a hat-shaped part in the periplasm (of diderms) or extracellular space (of monoderm Bacillus subtilis) and two cytoplasmic densities beneath, located ~10 nm below the IM (Fig. 2). In general, the periplasmic portion had a diameter of ~25 nm at its widest point at the outer leaflet of the IM, a diameter of ~10 nm at its top, and a height of ~12 nm.

The cytoplasmic densities were absent in the averages from three species: Pseudoalteromonas luteoviolacea, Hylemonella gracilis, and Bacillus subtilis. In P. luteoviolacea and B. subtilis, this could be due to the fact that the tomograms from these species, which were collected for other projects, were of lysed, not intact, cells. While lysing cells decreases their thickness, thereby increasing the quality of cryo-tomograms, it can also affect the integrity of macromolecular complexes, e.g., the flagellar motor (7). However, it is unclear why the cytoplasmic densities were also missing in H. gracilis, since these cryo-tomograms were of intact cells. In the averages from several other species, the cytoplasmic density did not resolve into two distinct sections (Fig. 2).

## MUTANT ANALYSIS

**FIG 3:**
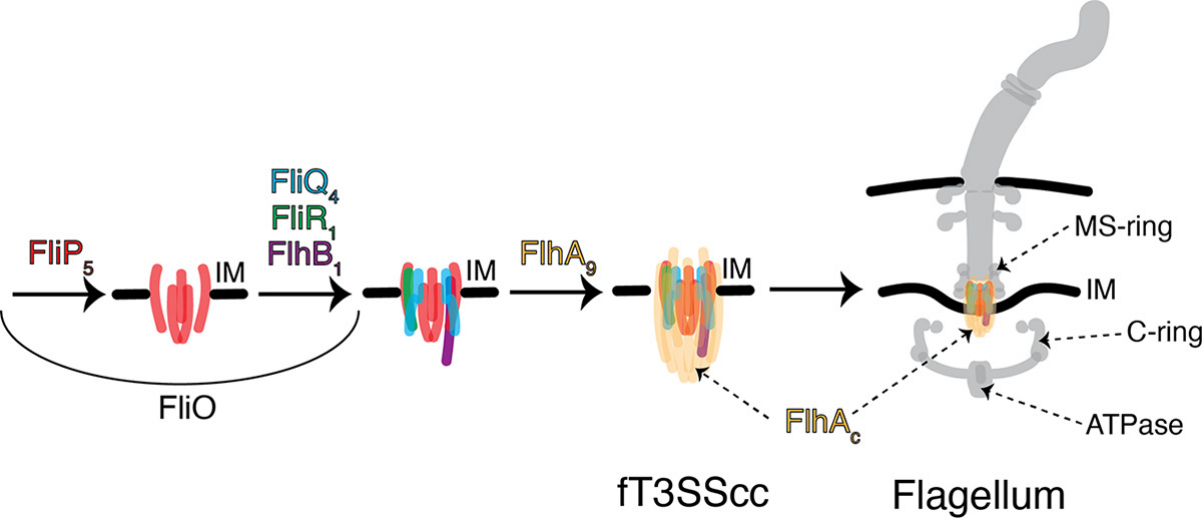
Schematic representation of the fT3SS assembly. The process starts with assembly of the fT3SScc proteins in the inner membrane (IM) and continues with the addition of the other components of the flagellum.

**FIG 4:**
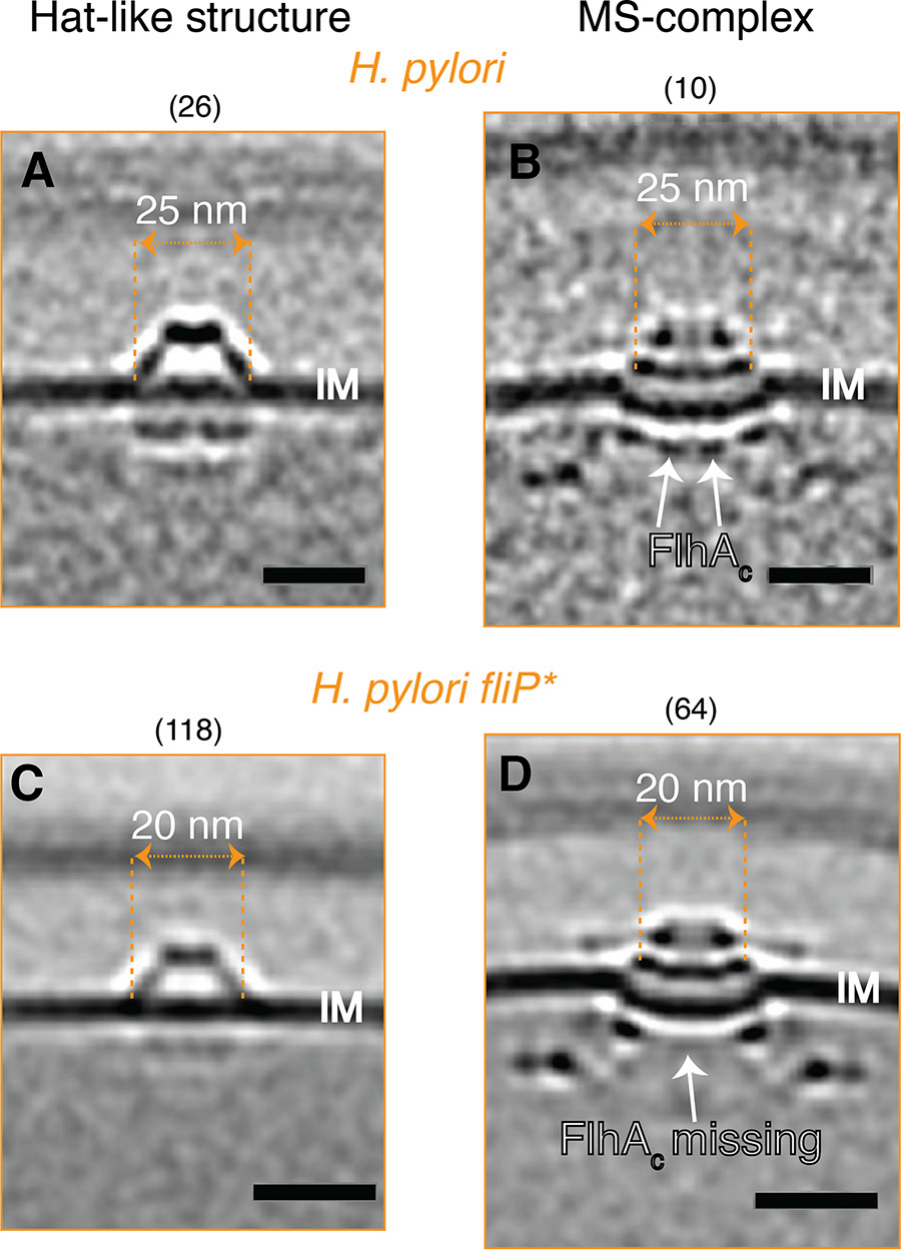
Comparison of the hat-like structure and MS-complexes in H. pylori strains. Central slices through subtomogram averages of the hat-like structures (A, C) and MS-complexes (B, D) of motile wild-type H. pylori (A, B) and nonmotile H. pylori fliP* (C, D). The diameters of the widest parts of the hat-like structures and the MS-rings are indicated. Numbers in parentheses indicate how many particles were used to produce the subtomogram average. (B, D) Adapted from (9). Scale bars represent 20 nm.

We identified the same novel structure in an H. pylori strain which contains a naturally occurring point mutation that disrupts the function of FliP (8), the platform on which other flagellar type III secretion system (fT3SS) proteins assemble (Fig. 3). We imaged this strain (henceforth referred to as H. pylori fliP*) in the course of a study of flagellar assembly (9). Curiously, the diameter of the hat-like density in H. pylori fliP* was reduced to only ~20 nm at its widest part, and the two cytoplasmic densities were missing (Fig. 4A and C). These changes reminded us of the changes that we observed in flagellar intermediates in H. pylori fliP*. The flagellar membrane/supramembrane-ring complex (MS-complex) of H. pylori fliP* similarly lacks its two cytoplasmic densities, and the MS-ring (FliF) adopts a smaller diameter (~20 nm) compared to that of motile H. pylori (~25 nm) (Fig. 4B and D). In the MS-complex, the two cytoplasmic densities, which also appear ~10 nm below the IM, are known to be formed by the C-terminal domain of the fT3SS protein FlhA (FlhA_C_), which forms a nonameric ring (Fig. 3 and 4B) (10). The similar dimensions of the hat-like structure and the MS-complex as well as the comparable changes that occur in H. pylori fliP*suggested to us that the hat-like structure is related to the fT3SS and that its cytoplasmic densities could also be formed by FlhA_C_.

The fT3SS consists of a cytoplasmic part containing an ATPase and an IM-embedded part known as the core complex (fT3SScc). The fT3SScc is comprised of five proteins (FliP, FliQ, FliR, FlhB, and FlhA), with another protein, FliO, being required for assembly but not forming part of the complex (11, 12). Initially, FliP forms a pentameric platform on which FliQ, FliR, and FlhB assemble to create a FliP_5_FliQ_4_FliR_1_FlhB_1_ subcomplex upon which an FlhA ring is built (13) (Fig. 3). To explore our hypotheses that the hat-like structure is related to the fT3SS and that its cytoplasmic densities are formed by FlhA_C_, we further mined our database for mutants lacking other fT3SScc proteins in species in which we had identified the hat-like structure.

**FIG 5:**
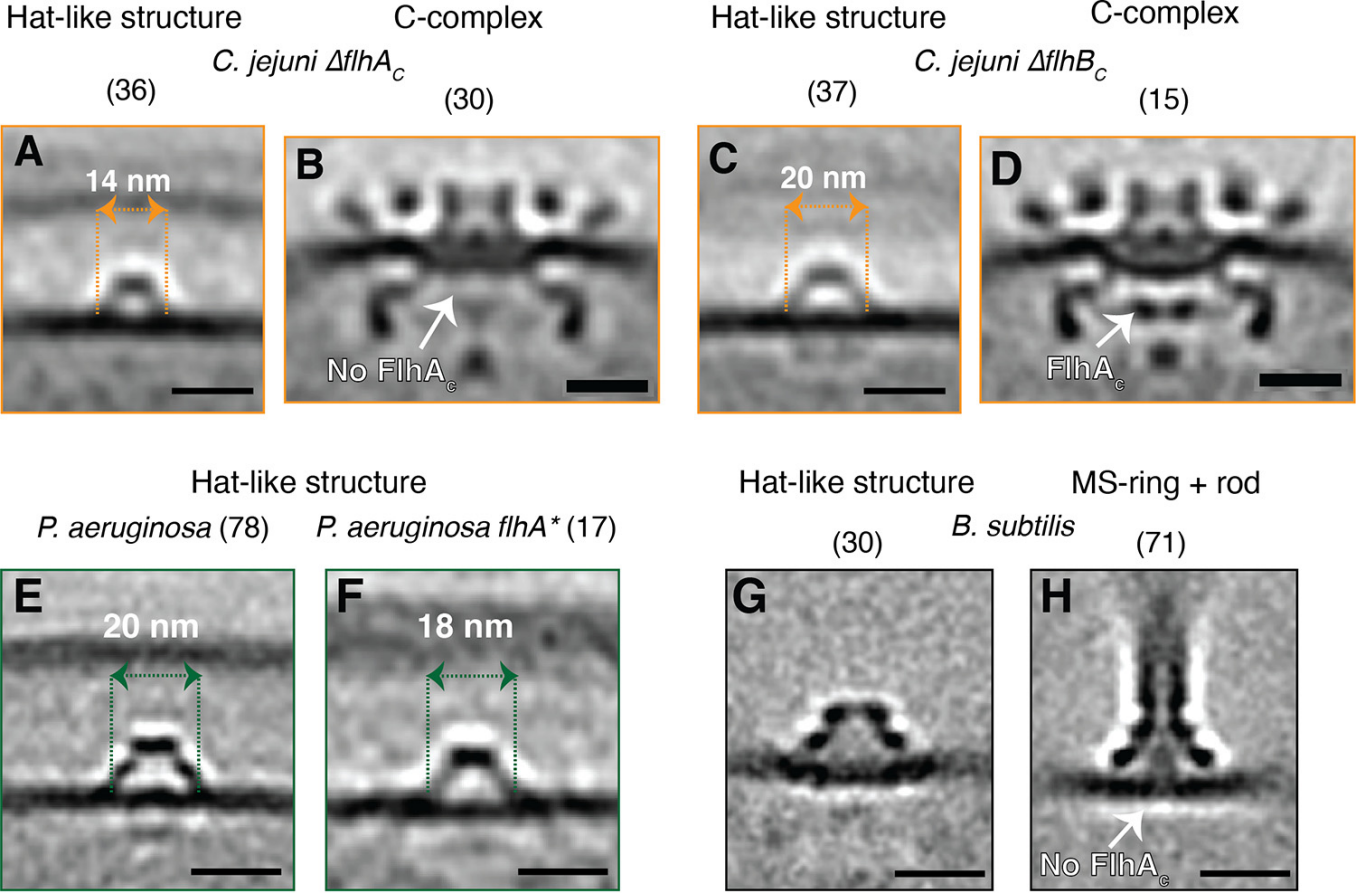
Comparison of the hat-like structure and flagellar subcomplexes in fT3SScc mutants and lysed cells. (A–D) Central slices through subtomogram averages of the hat-like structure (A, C) and flagellar C-complex (B, D) in C. jejuni Δ*flhA_c_* (A, B) and Δ*flhB_c_* (C, D) cells. (E, F) Central slices through subtomogram averages of the hat-like structure in P. aeruginosa wild-type (E) and flhA* (F) cells. (G, H) Central slices through subtomogram averages of the hat-like structure (G) and a flagellar subcomplex constituting the MS-ring and rod (H) from lysed B. subtilis cells. (H) Adapted from (7). Numbers in parentheses indicate how many particles were used to produce the subtomogram average. Scale bars represent 20 nm.

First, we examined Campylobacter jejuni mutants lacking the C-terminal domains of FlhA (Δ*flhAc*) and FlhB (Δ*flhBc*) (10). In the Δ*flhAc* cells, compared to wild-type cells, the periplasmic hat-like part was again smaller in diameter, and the two cytoplasmic densities again disappeared, supporting our hypothesis that they are formed by FlhA_C_ (Fig. 5A). Of course, it is possible that FlhA_C_ does not directly constitute the cytoplasmic densities but rather regulates the localization (or expression) of another protein or group of proteins that does. Such a regulatory role has been identified for another fT3SScc protein, FliO, which is responsible for the optimal expression of other flagellar genes (14). The Δ*flhAc* mutant arrests flagellar assembly at the C-complex (constituting the MS-ring, the fT3SS, the cytoplasmic [C-] ring, and other associated periplasmic components [9]), and we again observed that this intermediate lacked the cytoplasmic densities of FlhA_C_ (Fig. 5B). In contrast, in Δ*flhBc* cells, the hat-like structure was indistinguishable from the wild-type complex, both in diameter and in the presence of the associated cytoplasmic densities (Fig. 5C), and the cytoplasmic FlhA_C_ densities were also present in the flagellar C-complex (Fig. 4D). This lack of a visible difference was not surprising, since, unlike the large pentameric FliP ring or the nonameric FlhA ring, FlhB is a small protein present in a monomeric form in the fT3SScc. To confirm the generality of the relationship between the fT3SScc and the hat-like complex, we imaged an *flhA* mutant of P. aeruginosa (flhA*, obtained from a transposon insertion mutant library). Here, also, the hat-like structure appeared smaller in size (18 nm versus 20 nm) and lacked clear cytoplasmic densities compared to that of the wild-type cells (Fig. 5E and F), although the number of identified structures in the flhA* mutant was fewer than that of wild-type cells and produced a poorer-quality subtomogram average.

Another similarity between the hat-like structure and the flagellar system was the absence of the cytoplasmic densities in both structures in the cryo-tomograms of lysed cells. Recently, we showed that cell lysis can lead to the loss of the C-ring and FlhA_C_ densities in flagellar motors (7). In cryo-tomograms of lysed Bacillus subtilis, in which the hat-like structure lacked its two cytoplasmic densities, we identified a flagellar subcomplex consisting of the MS-ring and rod but lacking the cytoplasmic FlhA_C_ densities (Fig. 5G and H). Moreover, the MS-ring in this flagellar subcomplex looked morphologically similar (at the resolution of our subtomogram averages) to the hat-like structure (Fig. 5G and H).

**FIG 6:**
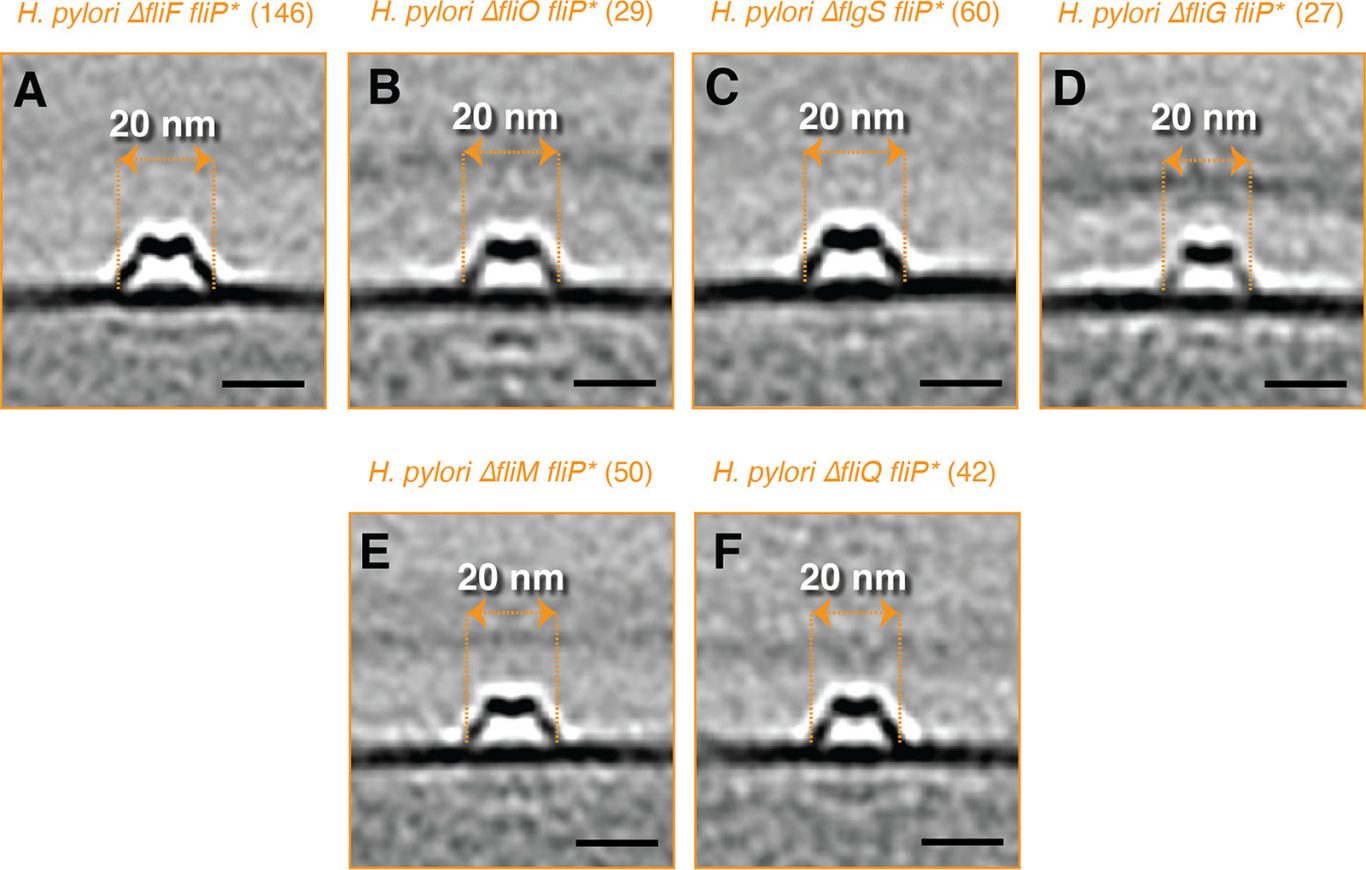
The effect of various flagellar-related mutations on the hat-like structure in H. pylori fliP*. (A–F) Central slices through subtomogram averages of the hat-like structures in the indicated mutants of H. pylori. Numbers in parentheses indicate how many particles were used to produce the subtomogram average. Scale bars represent 20 nm.

These observations strengthened our hypothesis that the hat-like structure is related to the fT3SS, and we speculated that the periplasmic hat may be formed by the flagellar MS-ring protein, FliF, which assembles on the fT3SScc during flagellar assembly (Fig. 3) in a different, more closed conformation than that observed in the flagellar motor. We therefore generated and imaged a Δ*fliF* mutant in the H. pylori fliP* background. To our surprise, while we could not identify any MS-complexes in this mutant, the hat-like complex was still present, though again with a smaller diameter and lacking clear cytoplasmic densities (Fig. 6A). The periplasmic hat, therefore, cannot be FliF. Subsequently, we speculated that it could be FliO, which is believed to have a large periplasmic domain in H. pylori (14) and is required for fT3SScc formation. We produced and imaged a ΔfliO fliP* H. pylori mutant but still observed hat-like structures (again lacking the cytoplasmic densities and with a reduced width of ~20 nm at the base), ruling out FliO, as well (Fig. 6B).

From our study of flagellar assembly, we had cryo-tomograms of several flagellar-related mutants of H. pylori: ΔflgS fliP*, ΔfliM fliP*, ΔfliG fliP*, and ΔfliQ fliP*. These mutants remove an additional fT3SScc protein (Δ*fliQ*), the C-ring proteins (Δ*fliM* and Δ*fliG*), or the tyrosine kinase responsible for the expression of the class II flagellar genes (Δ*flgS*) (15), and they were all constructed in the fliP* background to capture early flagellar intermediates (9). In all of these mutants, we observed hat-like structures, again with reduced diameter (~20 nm at the widest part), with missing or less well-resolved cytoplasmic densities (Fig. 6C–F). These differences were not due to decreased resolution, since in each case, more particles were averaged than from wild-type cells (see numbers above, subtomogram averages). We also investigated whether any of the H. pylori mutants affected the abundance of the hat-like structure but found no obvious correlation between the strain and the number of hat-like structures per cell (Fig. S2). While H. pylori fliP* cells had more structures per cell than did motile cells, other mutants, namely, ΔfliM fliP*, ΔfliO fliP*, and ΔfliQ fliP*, had fewer. Interestingly, the hat-like structure was still present in two mutants that did not contain visible flagellar intermediates, ΔfliG fliP* and ΔfliF fliP* (Fig. S2). Taken together, our observations suggest that while the cytoplasmic densities of the novel complex are likely FlhA_C_, the periplasmic density is not formed by FliF or other fT3SScc proteins.

## CORRELATION WITH FT3SS But Not With NF-T3SS

Next, we examined whether the hat-like structure is also related to the nonflagellar (NF-) T3SS. Bioinformatics analysis has shown that the NF-T3SS evolved from the flagellar system through a series of gene deletions, initially producing a machine thought to be involved in secreting proteins across the IM, an extant example of which is present in Myxococcales (16). Subsequently, the acquisition of an outer membrane-associated secretin resulted in the contact-dependent NF-T3SS (the injectisome) (16). First, we examined Myxococcus xanthus, which has NF-T3SS genes but no gene encoding a secretin, but found no hat-like structures in 95 cryo-tomograms (Fig. S3). Next, we examined Prosthecobacter vanneervenii, which has genes for the NF-T3SS and a secretin, but found no hat-like structures in 91 cryo-tomograms (Fig. S3). Enteropathogenic Escherichia coli (EPEC) has both the NF-T3SS injectisome and the fT3SS (17). In our database, we have cryo-tomograms of an EPEC strain that has the core complex proteins of the NF-T3SS (*escR*, *escS*, *escT*, *escU*, and *escV*) but lacks the other components of the injectisome and the fT3SScc (see Materials and Methods). We identified no hat-like structures in 200 cryo-tomograms of this strain (Fig. S3). Finally, as a negative-control, we investigated bacterial species that do not have any T3SS-related genes (neither fT3SS nor NF-T3SS). We examined Flavobacterium johnsoniae (206 cryo-tomograms), Coxiella burnetii (149 cryo-tomograms), and Amoebophilus asiaticus (9 cryo-tomograms) but did not identify any hat-like structures in any of these species (Fig. S3). Together, these results suggest that the hat-like structure is specifically related to the fT3SS (Table 1).

**TABLE 1:** Summary of the species investigated in this study, indicating the presence (+) or absence (−) of fT3SS genes, NF-T3SS genes, and hat-like structures in each

Species name	fT3SS genes	NF-T3SS genes	Hat-like structures
Helicobacter pylori	+	−	Yes
Xanthomonas citri	+	−	Yes
Vibrio harveyi	+	−	Yes
Vibrio fischeri	+	−	Yes
Vibrio cholerae	+	−	Yes
Pseudoalteromonas luteoviolacea	+	−	Yes
Pseudomonas flexibilis	+	−	Yes
Legionella pneumophila	+	−	Yes
Escherichia coli	+	−	Yes
Hyphomonas neptunium	+	−	Yes
Agrobacterium tumefaciens	+	−	Yes
Azospirillum brasilense	+	−	Yes
Brucella abortus	+	−	Yes
Helicobacter hepaticus	+	−	Yes
Campylobacter jejuni	+	−	Yes
Hylemonella gracilis	+	−	Yes
Salmonella enterica	+	+	Yes
Proteus mirabilis	+	+	Yes
Pseudomonas aeruginosa	+	+	Yes
Bacillus subtilis	+	+	Yes
Prosthecobacter vanneervenii	−	+	No
*EPEC* ^*a*^	−	+	No
Myxococcus xanthus	−	+	No
Flavobacterium johnsoniae	−	−	No
Coxiella burnetii	−	−	No
Amoebophilus asiaticus	−	−	No

a This EPEC mutant lacks all the NF-T3SS genes except the core complex ones and has all the flagellar genes except the fT3SScc ones.

## DISCUSSION

What could the function of the hat-like structure be? We do not know; we can only speculate. One possibility, for example, is that it plays a role in the assembly of early flagellar components. The fT3SScc is the first to assemble during flagellar biogenesis, followed by the MS- and C-rings (11, 12). Then, the process proceeds in a cooperative way in which the absence of a certain component prevents subsequent parts from assembling (18–20). However, we recently found that in an H. pylori fliP* strain, the MS-ring, which depends on Sec for secretion through the IM (21), can still assemble in the absence of the fT3SScc (9). One hypothesis for the function of the novel structure described here is that it is somehow involved in this process of early flagellar biogenesis. A physical association with the fT3SScc would explain the disappearance of the cytoplasmic densities and the smaller diameter of the periplasmic portion in the fliP* and *flhA* mutants. While bioinformatics analysis in the future might help to identify candidates for this structure, the fact that flagellar genes do not always cluster in the same way in all species (22) makes this challenging.

Another possibility is that this apparently ancient structure may have diverged to serve a function independent of flagellar assembly. Supporting an independent role, the hat-like structures did not show a preferred spatial distribution in the cell, even in species with polar flagella. It is also possible that the structure serves different functions in different species. This could explain the structural variations we observed, such as the absence of the cytoplasmic densities in H. gracilis. Whatever the function of the hat-like complex, it joins the already rich repertoire of the (f)T3SS, which has roles in flagellar motility and protein translocation and was recently implicated in membrane nanotube formation (23).

## MATERIALS AND METHODS

### Strains and growth conditions.

E. coli cells were grown as described (24). X. citri cells were grown in 2xTY medium for 14 h to stationary phase. V. cholerae, V. harveyi, and V. fischeri were grown as described (25). P. luteoviolacea were grown as described (4, 26). P. mirabilis were grown as described (27). P. aeruginosa were grown in LB medium at 37°C overnight. The P. aeruginosa flhA* mutant was obtained from a transposon library (mutant number 3296 from the nonredundant library http://pa14.mgh.harvard.edu/cgi-bin/pa14/downloads.cgi) from Dianne Newman’s lab at Caltech. L. pneumophila were grown as described (28). S. enterica were grown as described (29). P. flexibilis were grown in lactose growth medium. H. neptunium were grown to exponential phase in PYE medium. A. tumefaciens wild-type cells with a plasmid-borne VirC1-GFP translational fusion under control of the VirB promoter were grown in AB medium with 150 to 300 μg/mL of kanamycin. A. brasilense and B. abortus were grown as described (3). H. hepaticus ATCC 51449 and H. gracilis were grown as described (24, 30). C. jejuni and its mutants were grown as described (10, 29, 31). B. subtilis protoplasts and lysate were prepared with lysozyme, following protocol (32). A motile revertant H. pylori 26695 isolate was selected by serial passage in Brucella broth supplemented with 10% heat-inactivated fetal bovine serum at 37°C, 5% CO_2_ for 4 days until cultures reached an OD_600_ of ~0.4. Nonmotile H. pylori fliP* mutants were propagated on TSAII blood agar plates (BD Biosciences) at 37°C, 5% CO_2_ for either 24 or 48 h prior to collection with a sterile cotton swab for grid preparation. H. pylori mutants (ΔfliM fliP*, ΔfliO fliP*, ΔflgS fliP*, ΔfliG fliP*, ΔfliQ fliP*, and ΔfliF fliP*) were grown directly from glycerol stocks on sheep blood agar at 37°C with 5% CO_2_ for 48 h. Cells were either collected from the plate using a cotton swab, resuspended in PBS, then spun down and plunge-frozen directly, or passaged onto a new plate and allowed to grow for 24 h under the same conditions prior to collection and plunge-freezing. No difference could be discerned between the two samples by cryo-ET.

M. xanthus DK1622 cells were grown for 48 h in CTT medium at 30°C without antibiotics to OD_600_=0.7. F. johnsoniae strain CJ2618 was grown as described (4). P. vanneervenii cells were grown as described (33). A. asiaticus strain 452471 was grown as described (34). C. burnetii Nine Mile phase II clone 4 strain was grown in the laboratory of Robert Heinzen at the National Institute of Allergy and Infectious Diseases. EPEC cells strain 8612 (Δ*rorf1-escL* Δ*rorf3-mpc* Δ*escN-espF* Δ*flhBA* Δ*fliOPQR* Δ*minCD*) from a glycerol stock were grown in 2mL LB at 37°C in static conditions overnight. The next day, 1 mL of cells were spun down at 4,000 rpm for six minutes and concentrated 10 times. 3 μL of cells were spotted onto glow-discharged grids placed on DMEM high-glucose agar plates (1.5% agar) for 3 h. Then, the grids were washed twice in PBS and transferred to starvation-medium (DMEM without phenol red, vitamins, and amino acids) agar plates and incubated for 1 h at 37°C. Subsequently, the samples were washed with PBS and plunge-frozen.

### H. pylori mutagenesis.

Flagellar mutants were generated in the nonmotile H. pylori 26695 background as previously described (9, 35).

### Electron cryo-tomography sample preparation and imaging.

Sample preparation for cryo-ET imaging was done as described (20, 25, 36). All data were collected by the Jensen Lab at Caltech. For the total cumulative electron dose used for each tilt-series in each species, see Table 2.

**TABLE 2:** Total electron dose used for each tilt-series in each species

Species name	Class	Cumulative electron dose (e-/Å^2^)
Xanthomonas citri	Gammaproteobacteria	120
Vibrio harveyi	Gammaproteobacteria	160
Vibrio fischeri	Gammaproteobacteria	150
Vibrio cholerae	Gammaproteobacteria	160
Salmonella enterica	Gammaproteobacteria	200
Pseudoalteromonas luteoviolacea	Gammaproteobacteria	180
Proteus mirabilis	Gammaproteobacteria	160
Pseudomonas flexibilis	Gammaproteobacteria	100
Pseudomonas aeruginosa	Gammaproteobacteria	170
Legionella pneumophila	Gammaproteobacteria	100
Escherichia coli	Gammaproteobacteria	130
Hyphomonas neptunium	Alphaproteobacteria	180
Agrobacterium tumefaciens	Alphaproteobacteria	200
Azospirillum brasilense	Alphaproteobacteria	200
Brucella abortus	Alphaproteobacteria	160
Helicobacter hepaticus	Epsilonproteobacteria	200
Helicobacter pylori	Epsilonproteobacteria	120 to 130
Campylobacter jejuni	Epsilonproteobacteria	200
Hylemonella gracilis	Betaproteobacteria	75
Bacillus subtilis	Bacilli	160
Myxococcus xanthus	Deltaproteobacteria	200
Prosthecobacter vanneervenii	Verrucomicrobiae	180
Flavobacterium johnsoniae	Flavobacteria	100
Coxiella burnetii	Gammaproteobacteria	130
Amoebophilus asiaticus	Cytophagales	200
*EPEC*	Gammaproteobacteria	120

### Image processing and subtomogram averaging.

The three-dimensional reconstruction of the tilt-series was performed either automatically through the RAPTOR pipeline used in the Jensen Lab (5) or with the IMOD software package (37). Subtomogram averaging was done using the PEET program (38) with a 2-fold symmetrization applied along the particle *y*-axis. The numbers of particles included in each subtomogram average are indicated on the figures.
