# Supplementary material for: Discovery of a Novel Inner Membrane-Associated Bacterial Structure Related to the Flagellar Type III Secretion System
Source: J Bacteriol. 2022 Jul 18;204(8):e00144-22. doi: 10.1128/jb.00144-22 (PMC9380563; doi:10.1128/jb.00144-22)
Supplement: Supplemental file 1 — Fig. S1 to S3 and caption for Movie S1. Download jb.00144-22-s0001.pdf, PDF file, 6.4 MB [file jb.00144-22-s0001.pdf]

Supporting Figures for:

Discovery of a novel inner-membrane-associated bacterial structure related to the flagellar type III secretion system

Mohammed Kaplan[1], Catherine M. Oikonomou[1], Cecily R. Wood[2], Georges Chreifi[1], Debnath Ghosal[3], Megan J. Dobro[4], Qing Yao[1], Ritesh Ranjan Pal[5,6], Amit K. Baidya[7], Yuxi Liu[1], Stefano Maggi[1], Alasdair W. McDowall[1], Sigal Ben-Yehuda[5], Ilan Rosenshine[5], Ariane Briegel[8], Morgan Beeby[9], Yi-Wei Chang[10], Carrie L. Shaffer[2,11,12], Grant J. Jensen[1,13,*]

Affiliations

[1]Division of Biology and Biological Engineering, California Institute of Technology, Pasadena, CA 91125, USA

[2]University of Kentucky Department of Veterinary Science

[3]Division of Medicine, Dentistry and Health Sciences, University of Melbourne, Parkville, Victoria, Australia

[4]Hampshire College, Amherst, Massachusetts, USA

[5]Department of Microbiology and Molecular Genetics, Institute of Medical Research Israel-Canada, Faculty of Medicine, The Hebrew University of Jerusalem 91120, Jerusalem, Israel

[6] School of Biological Sciences, Indian Association for the Cultivation of Science, Kolkata, India

[7]Molecular Biophysics Unit, Indian Institute of Science, Bangalore 56001, Karnataka, India

[8]Leiden University, Sylvius Laboratories, Leiden, The Netherlands

[9]Department of Life Sciences, Imperial College London, South Kensington Campus, London SW7 2AZ, UK

[10]Department of Biochemistry and Biophysics, Perelman School of Medicine, University of Pennsylvania, Philadelphia, PA 19104, USA

[11]University of Kentucky Department of Microbiology, Immunology, and Molecular Genetics

[12]University of Kentucky Department of Pharmaceutical Sciences

[13]Department of Chemistry and Biochemistry, Brigham Young University, Provo, UT 84604, USA

[*]Corresponding author: grant_jensen@byu.edu

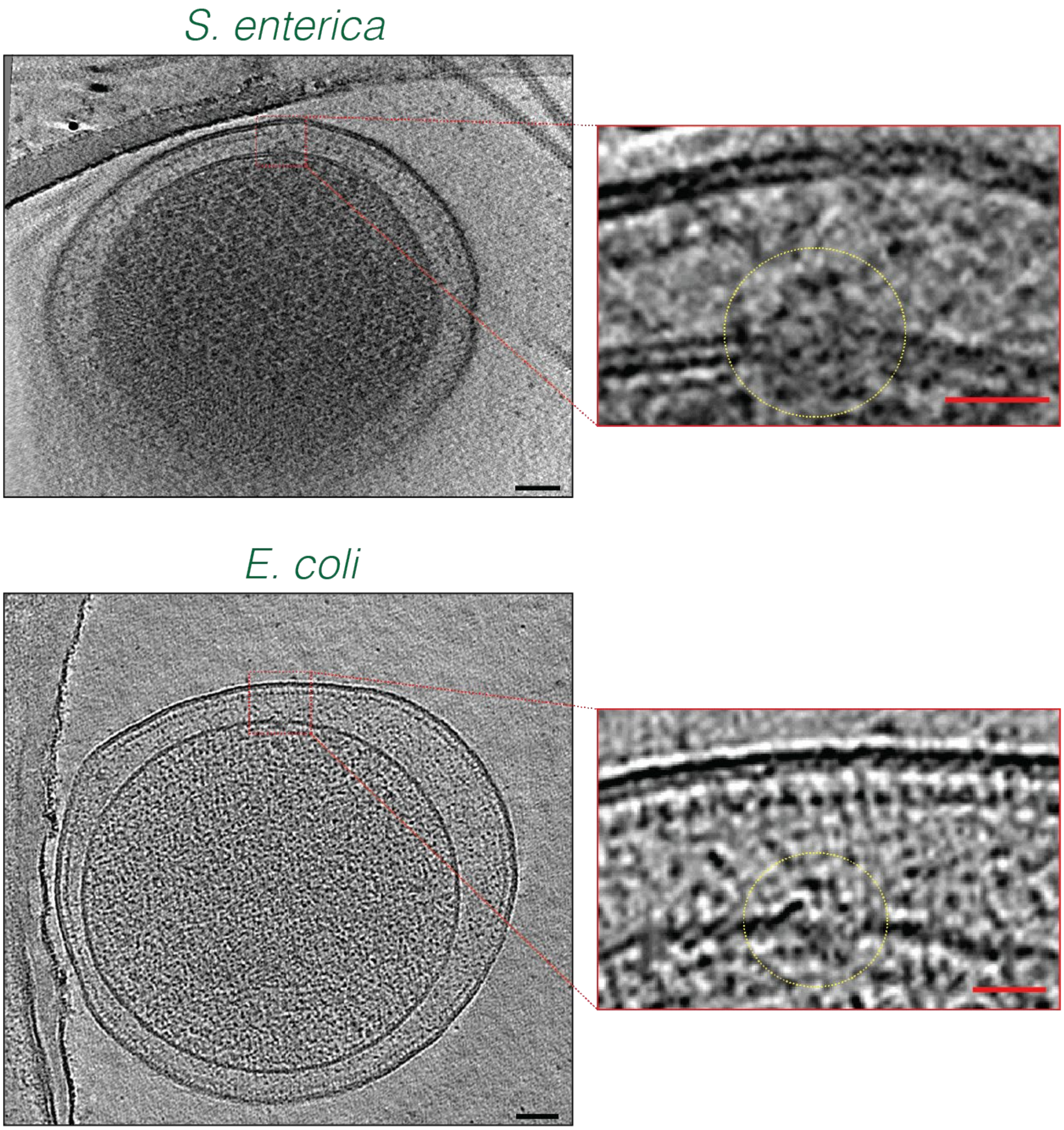
S. enterica
E. coli

32

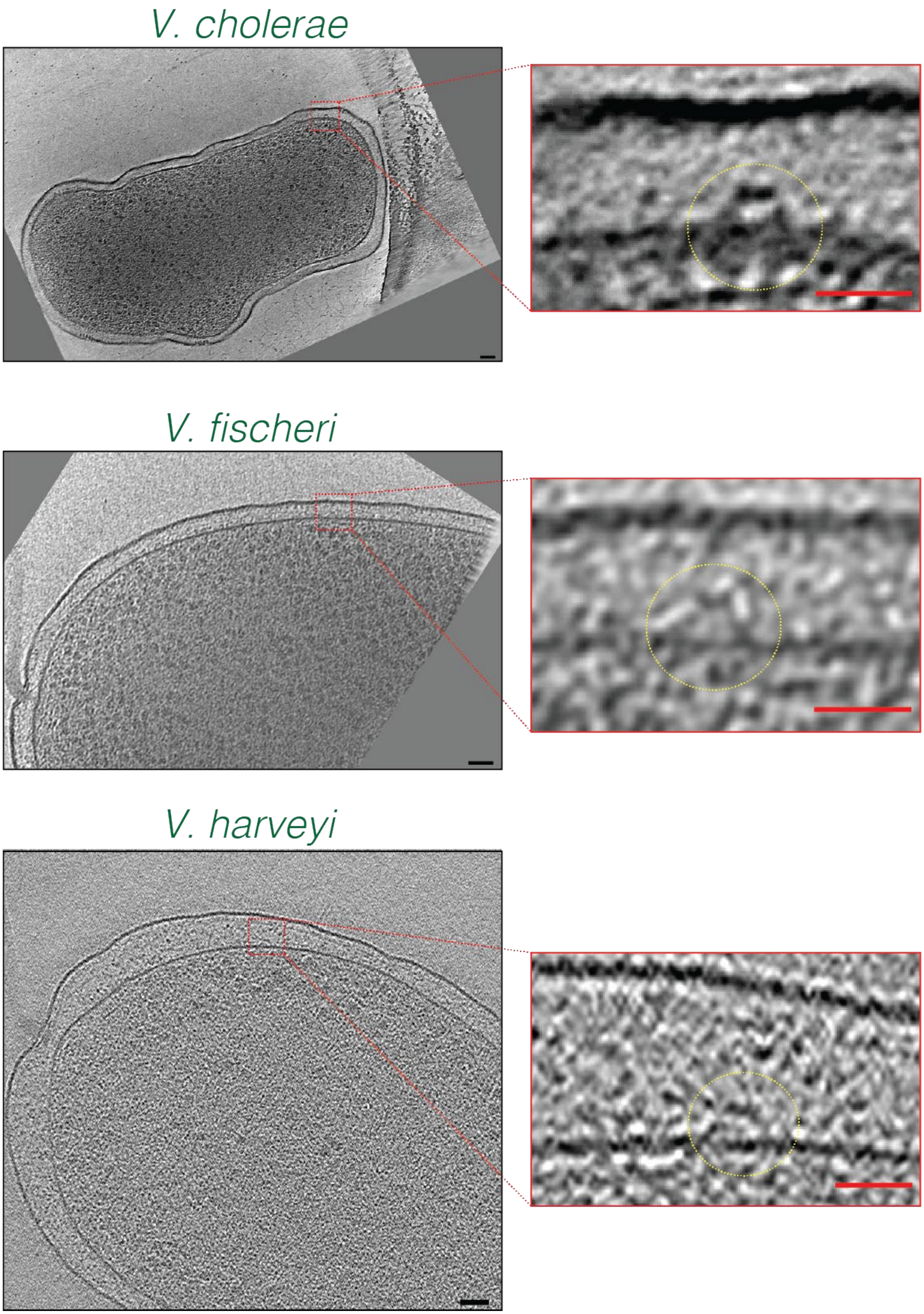

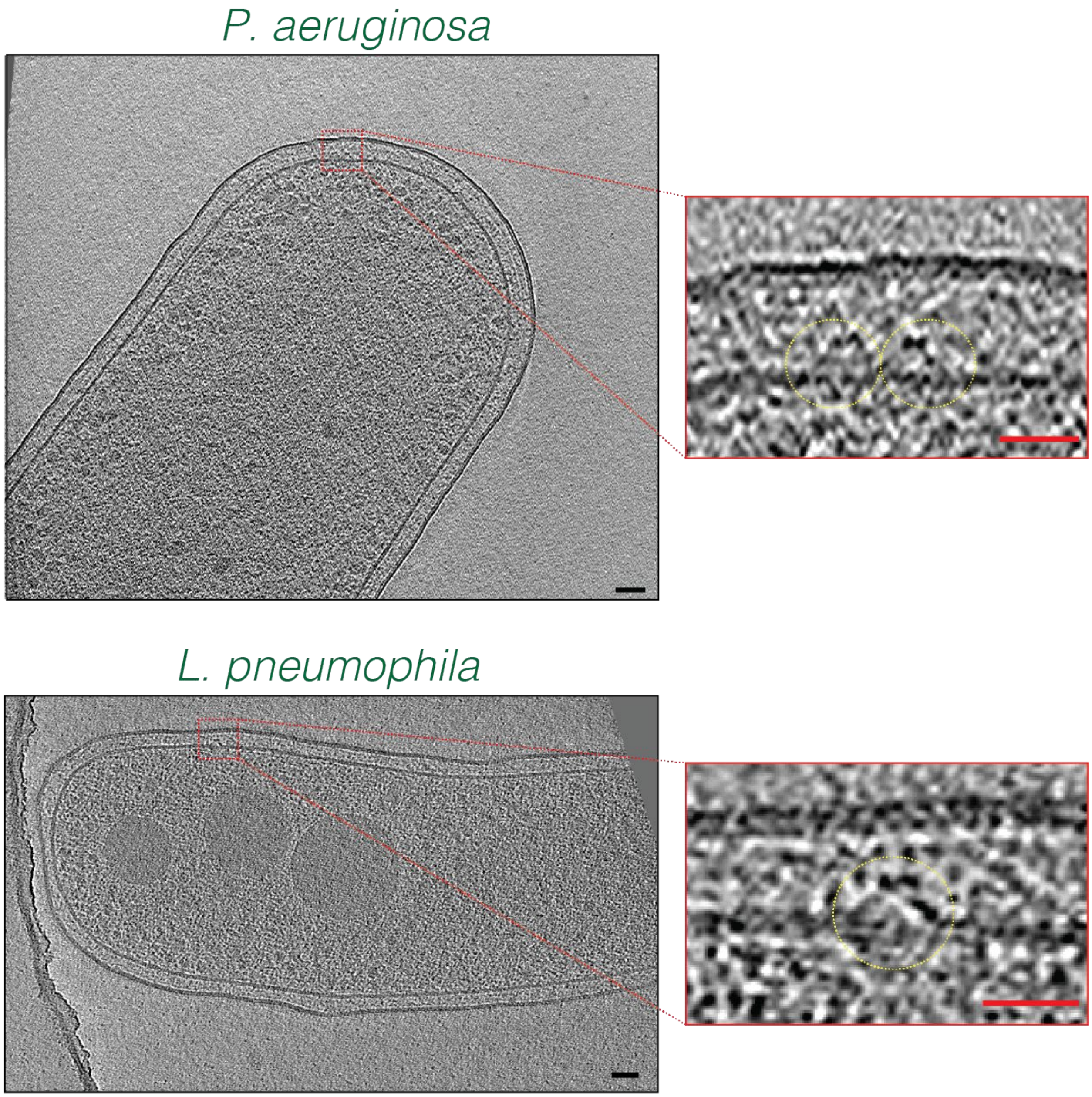
P. aeruginosa
L. pneumophila

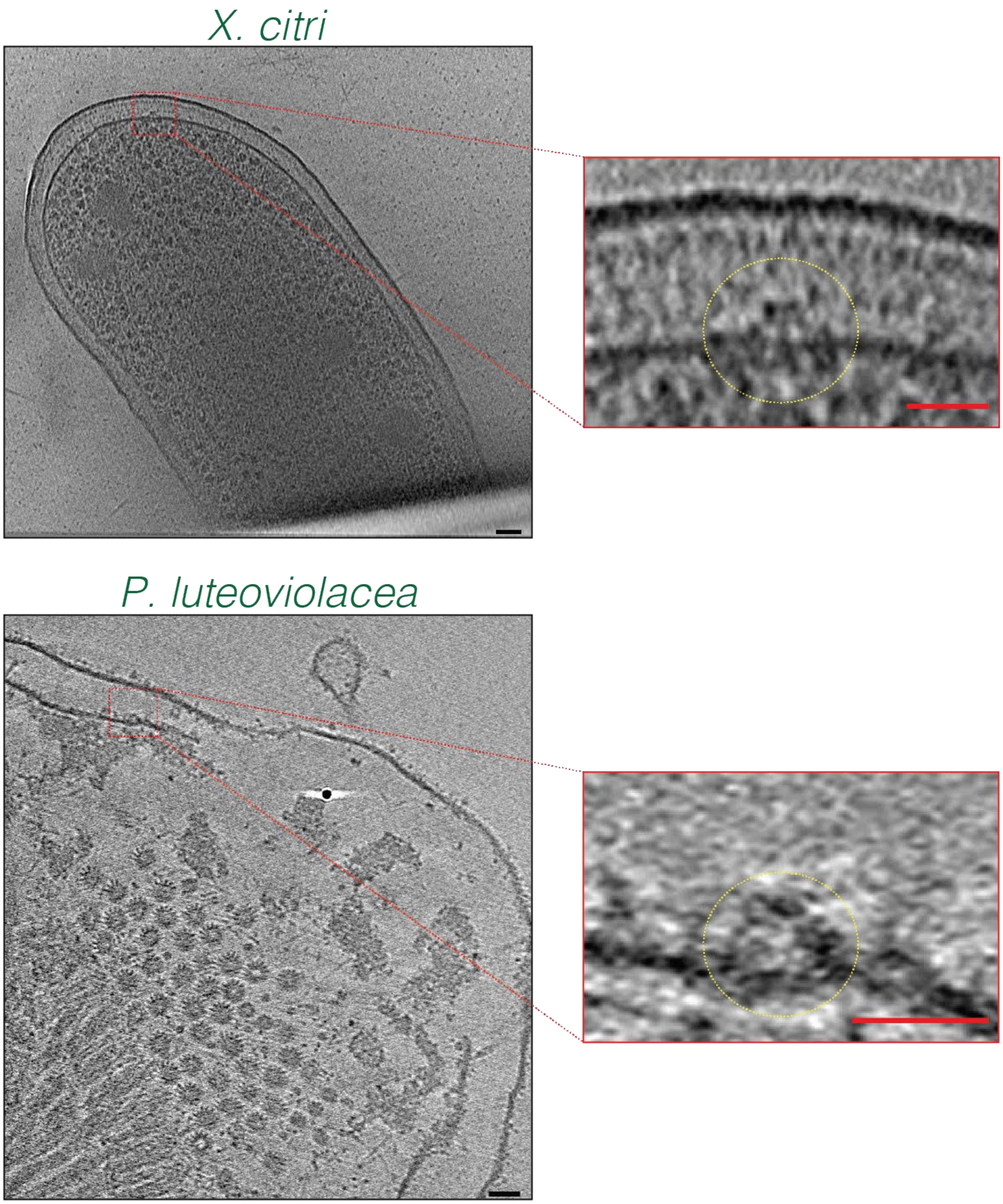
X. citri
P. luteoviolacea

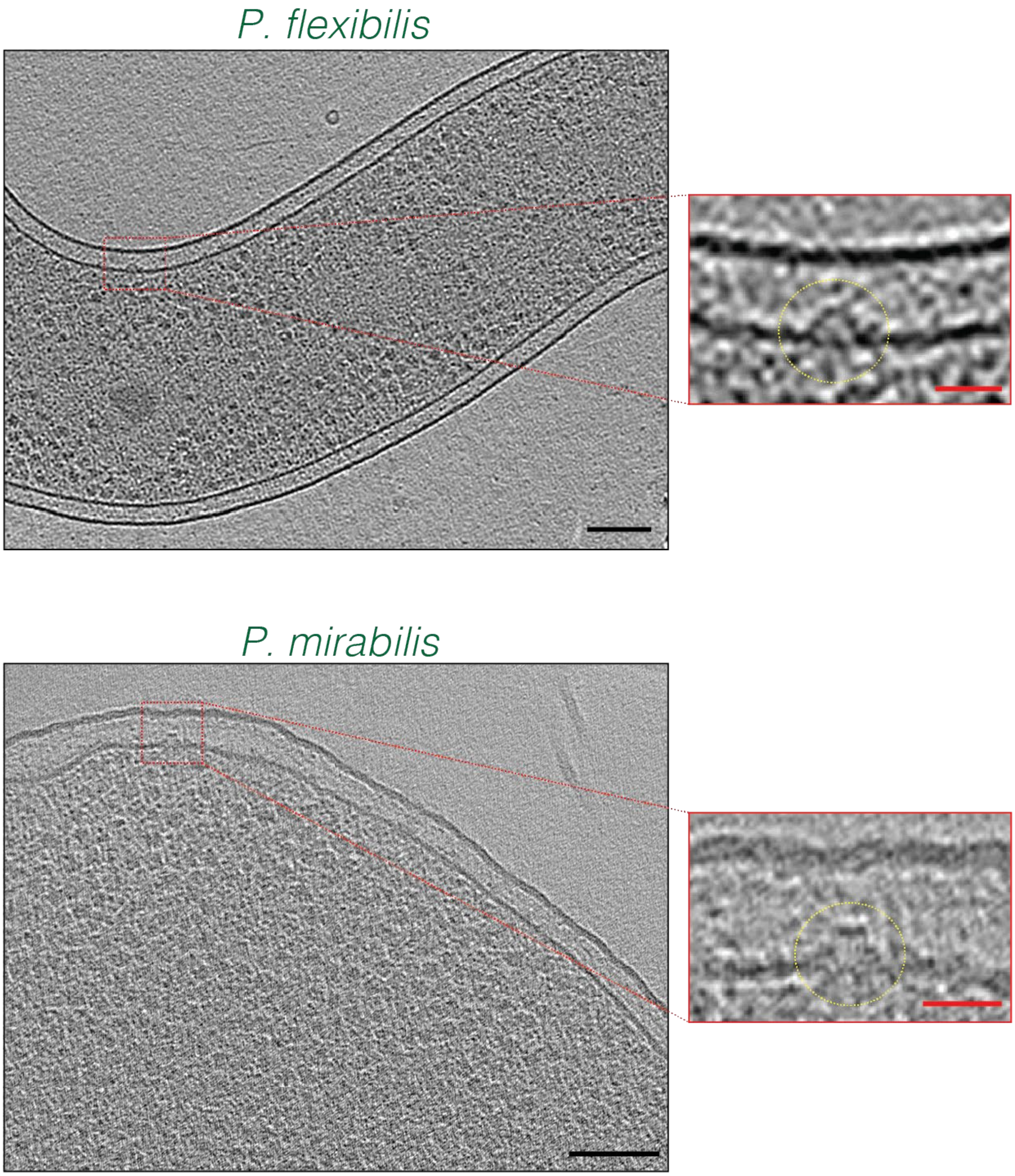
P. flexibilis
P. mirabilis

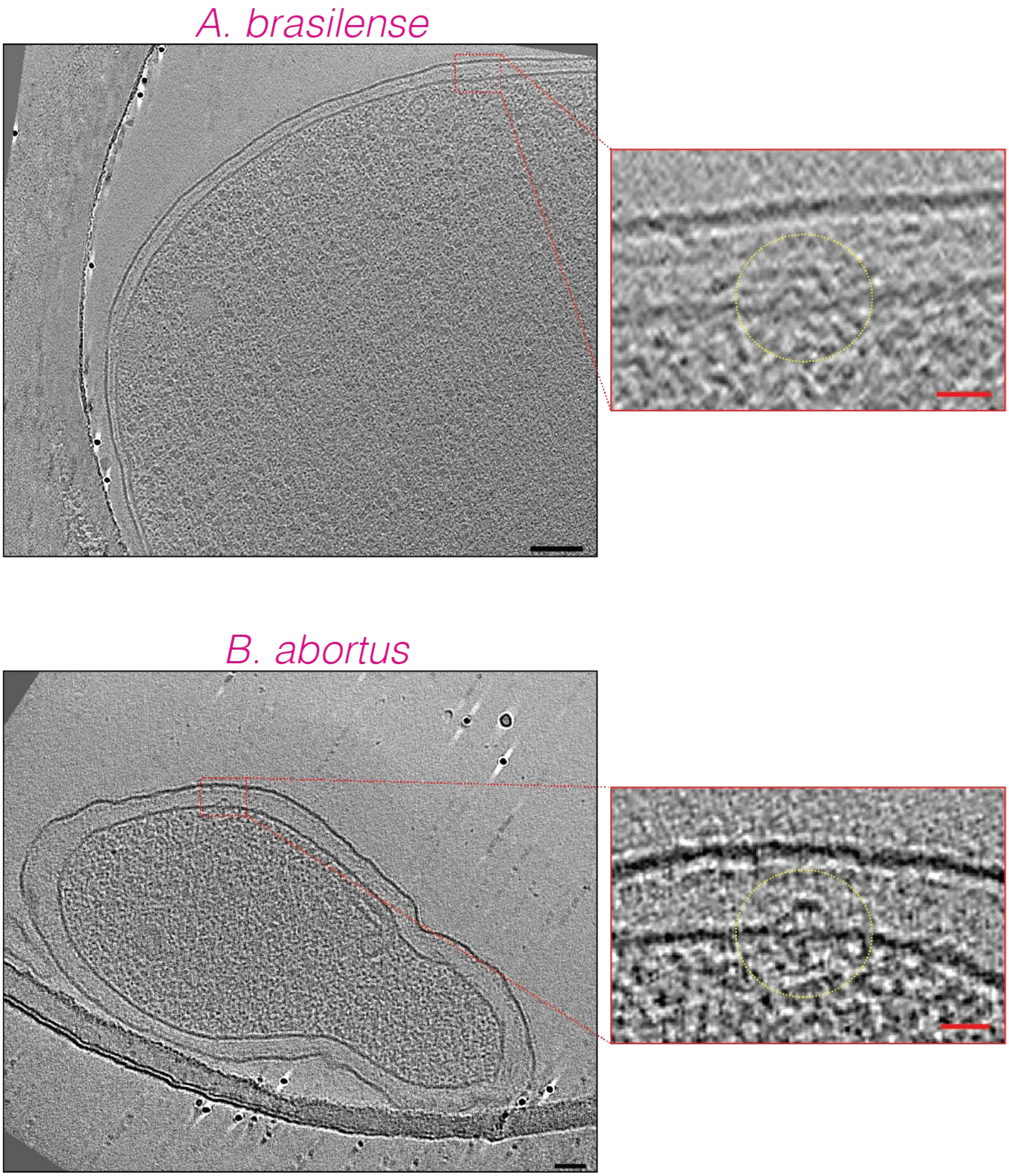
A. brasilense
B. abortus

A. tumefaciens

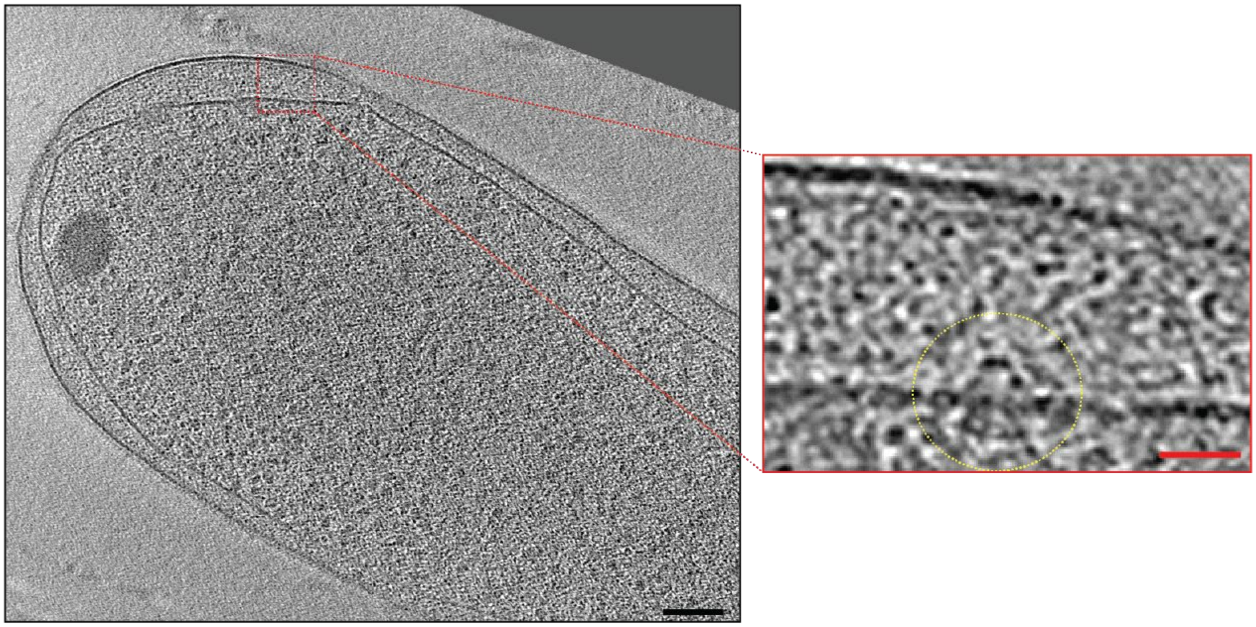

H. neptunium

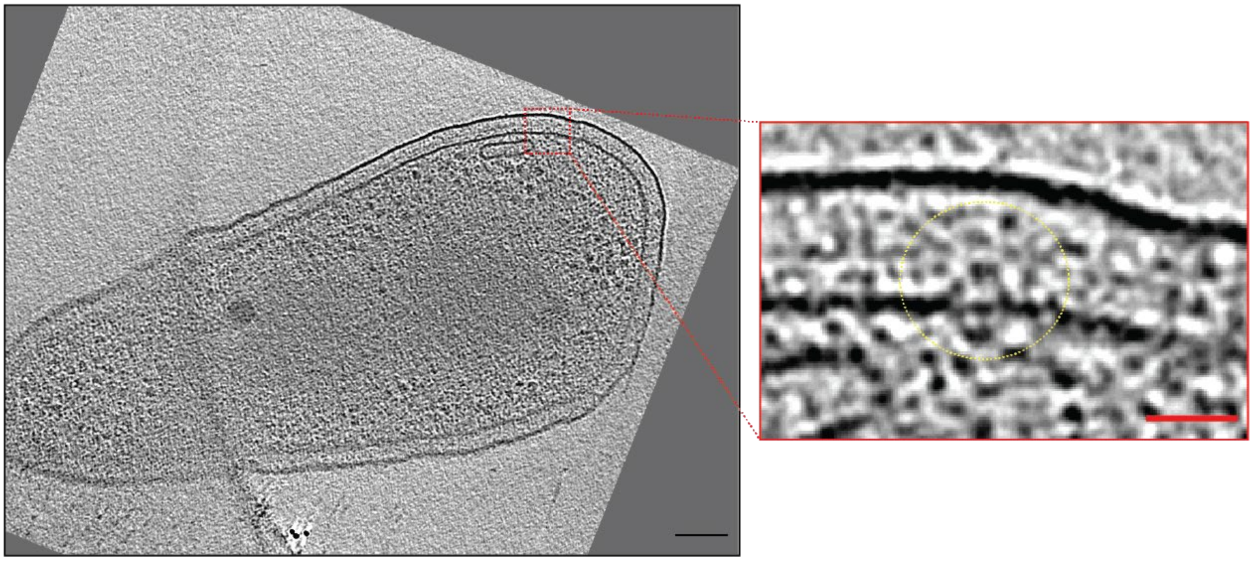

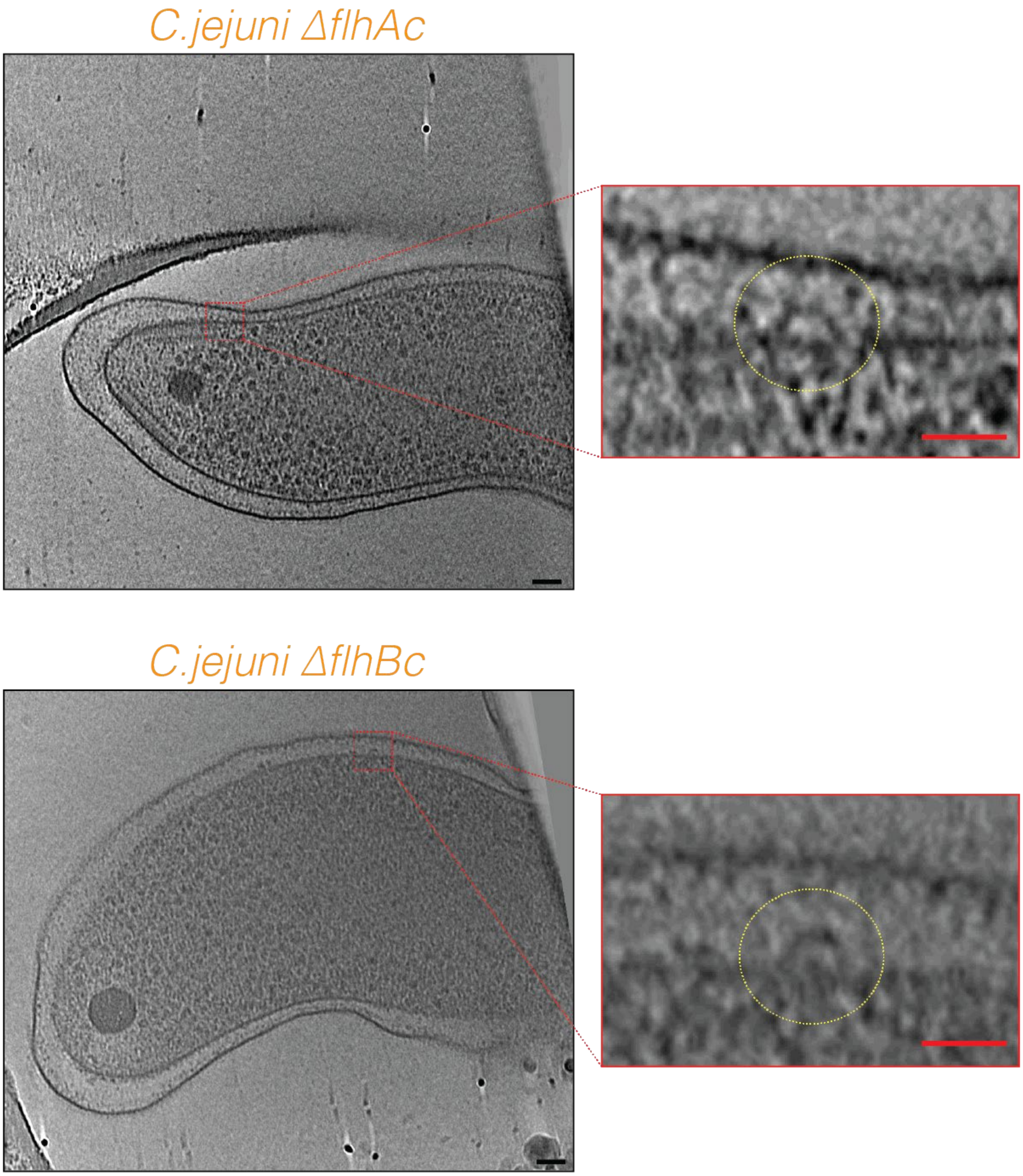
C.jejuni ΔflhAc
C.jejuni ΔflhBc

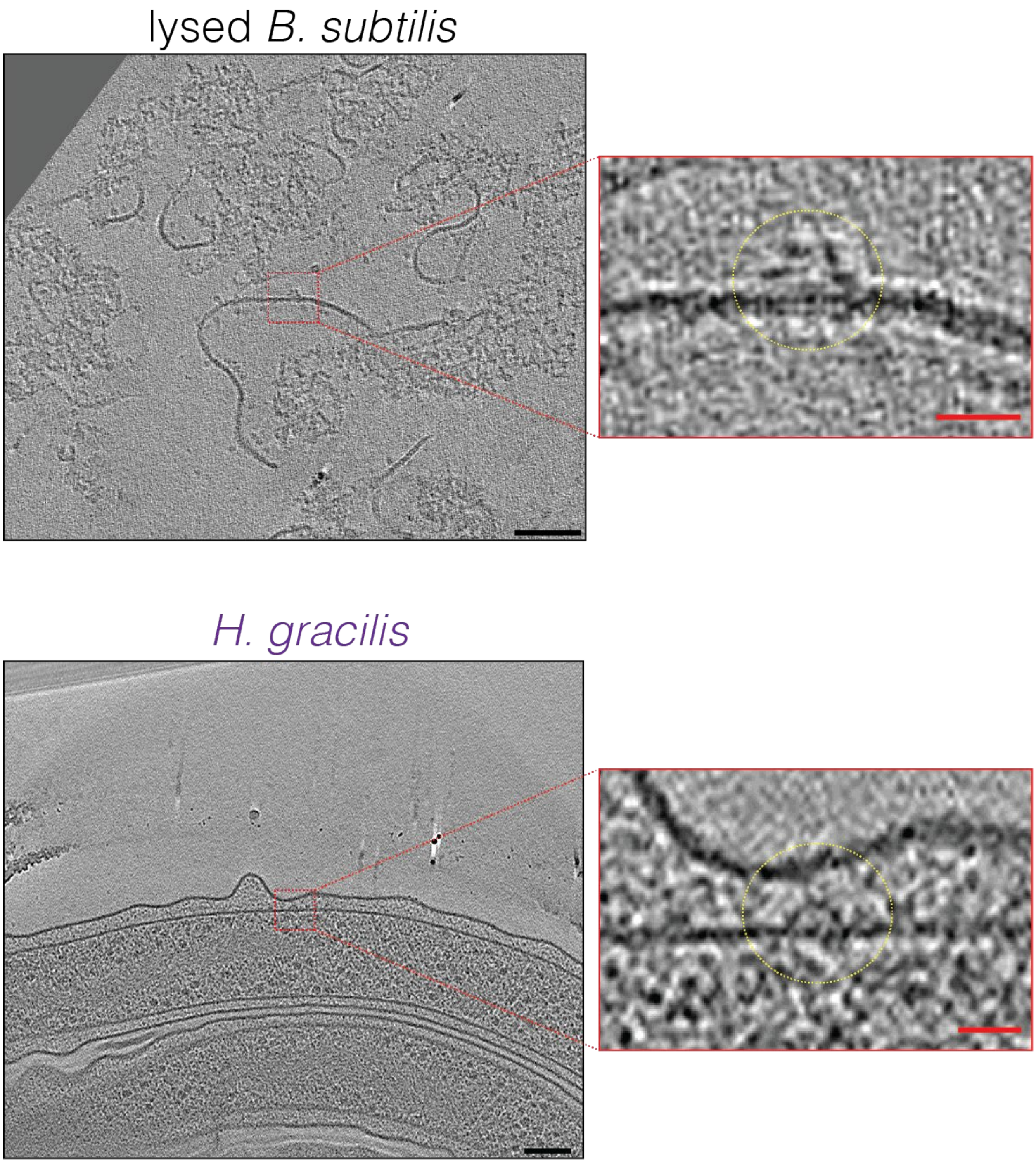
lysed B. subtilis
H. gracilis

Figure S1: The hat-like structure is widespread in various bacterial species. Slices through electron cryo-tomograms of various bacterial species highlighting the presence of hat-like complexes (yellow circles in the enlarged views). Black scale bars 100 nm, red scale bars 20 nm.

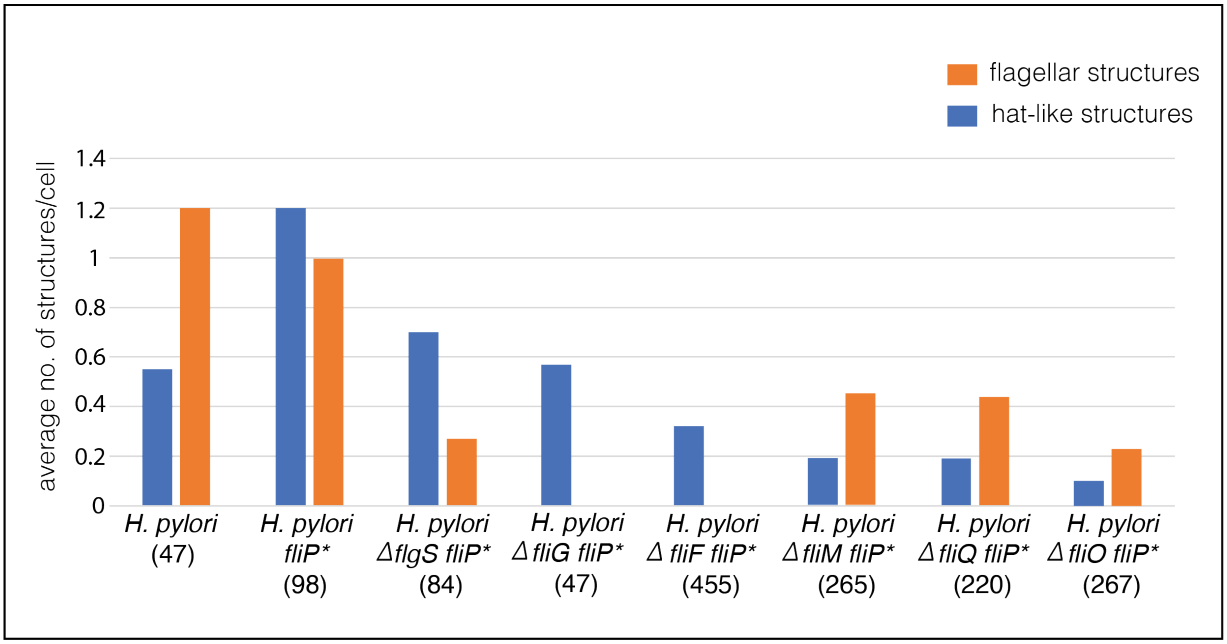

Figure S2: Abundance of hat-like and flagellar structures in motile *H. pylori* cells and various mutants. The bar chart shows the average number of hat-like structures and flagellar structures per cell in motile *H. pylori* and various mutants. The number of cells examined for each strain is indicated in parentheses. Flagellar structures include fully-assembled flagella and any intermediate assembly stage where the MS-ring is present. In two mutants (Δ*fliG fliP** and Δ*fliF fliP**), only hat-like structures were identified but no flagellar structures.

Myxococcus xanthus

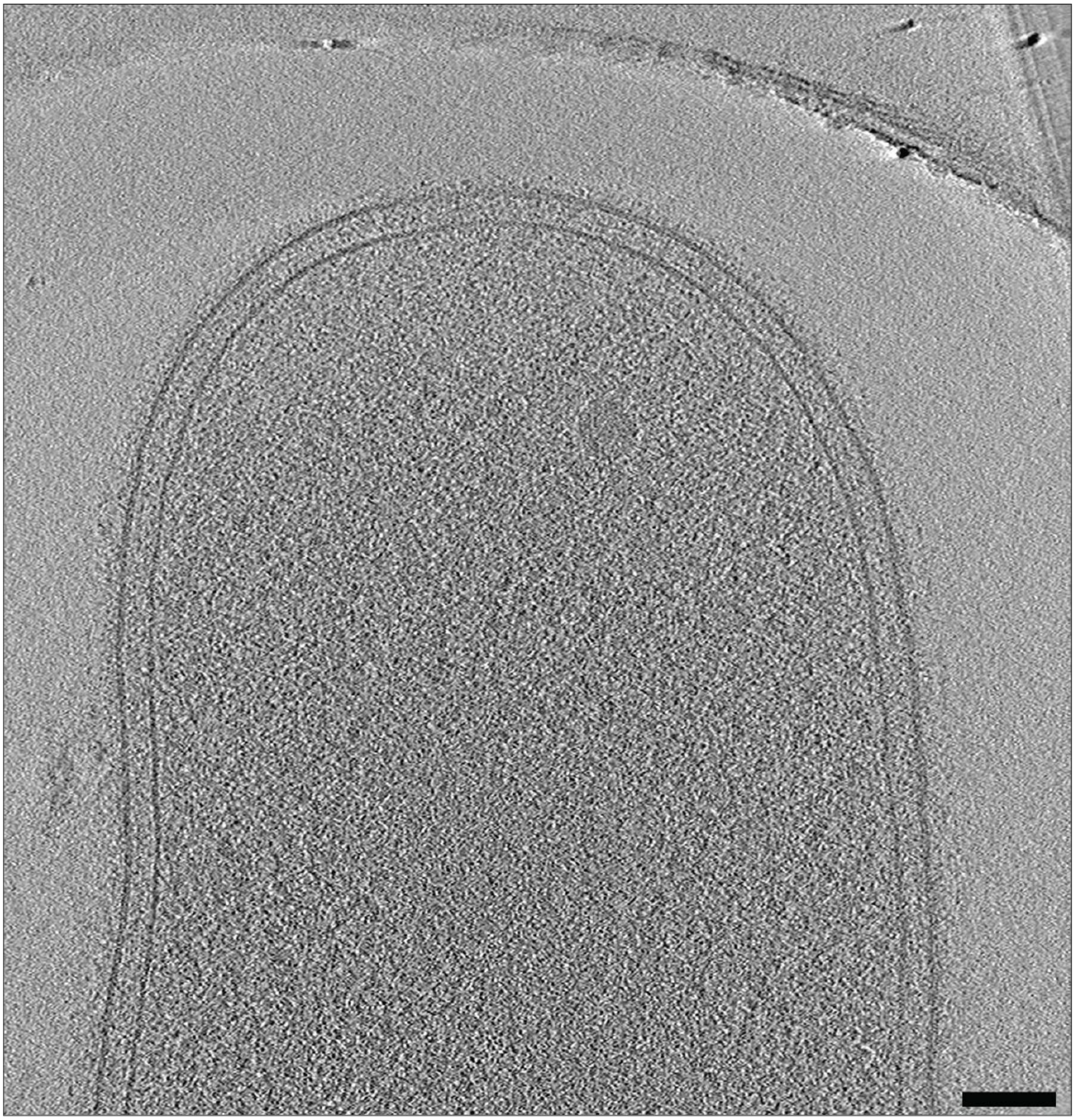

Prosthecobacter vanneervenii

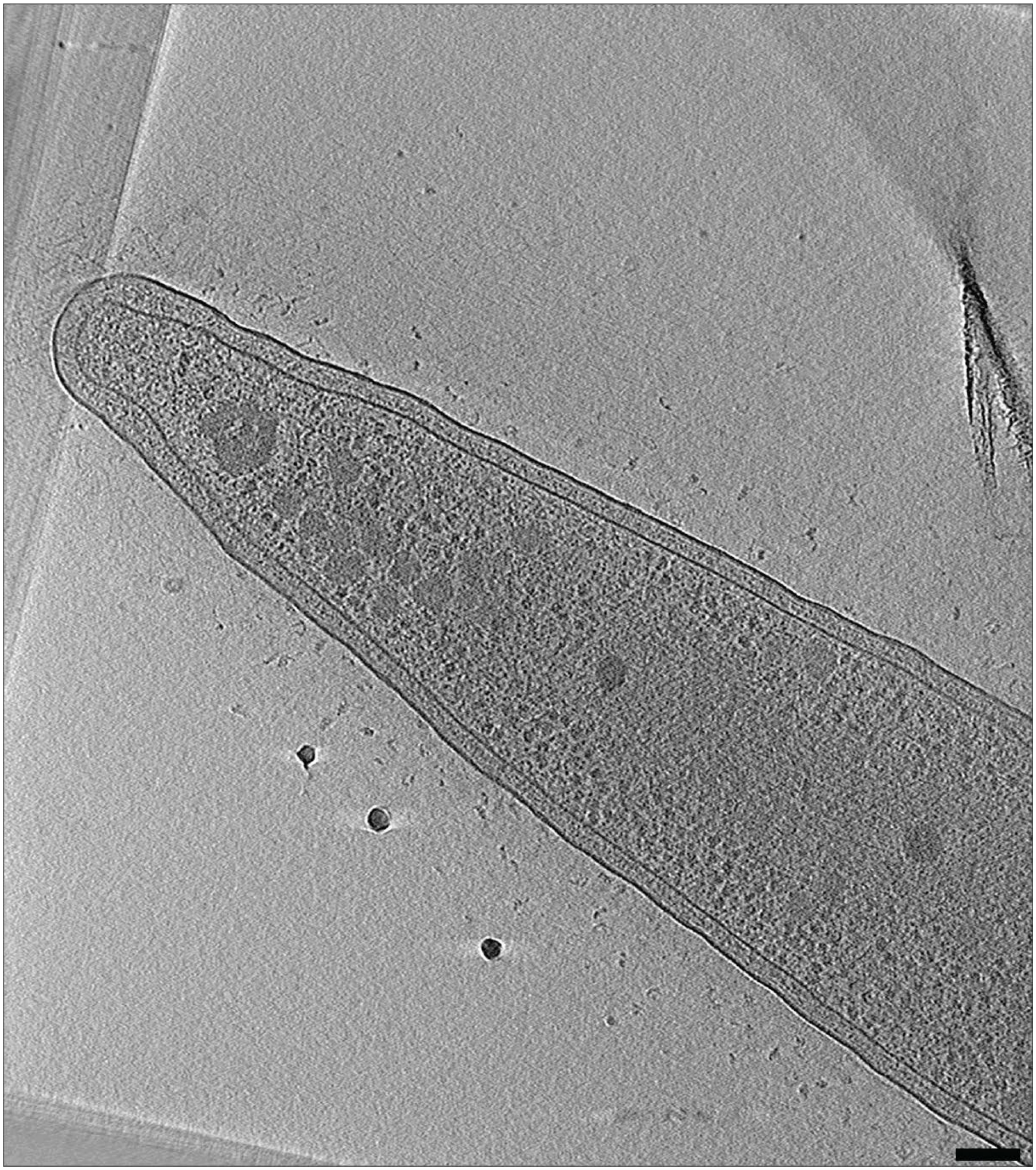

Enteropathogenic
Escherichia coli (EPEC)

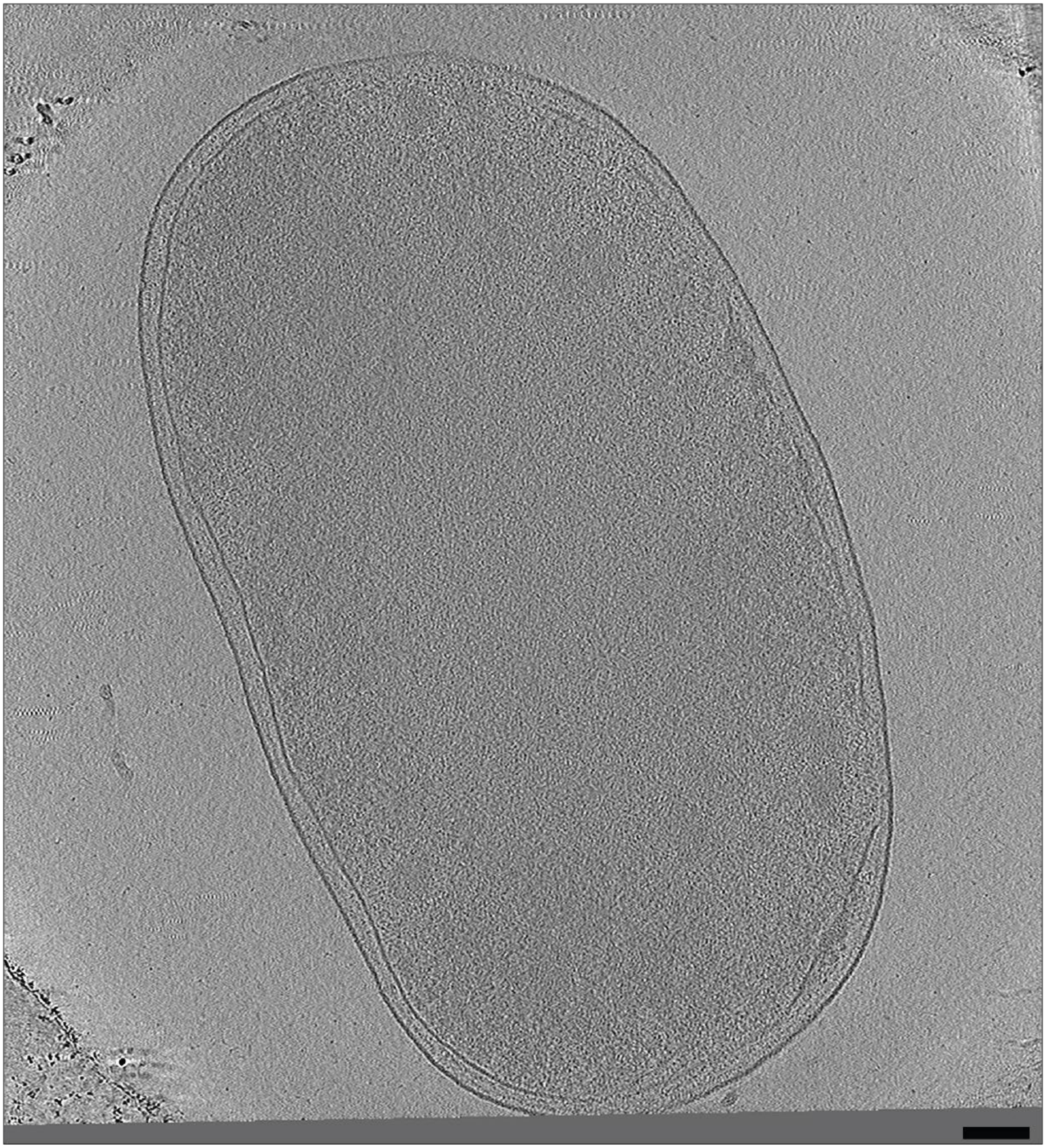

Flavobacterium johnsoniae

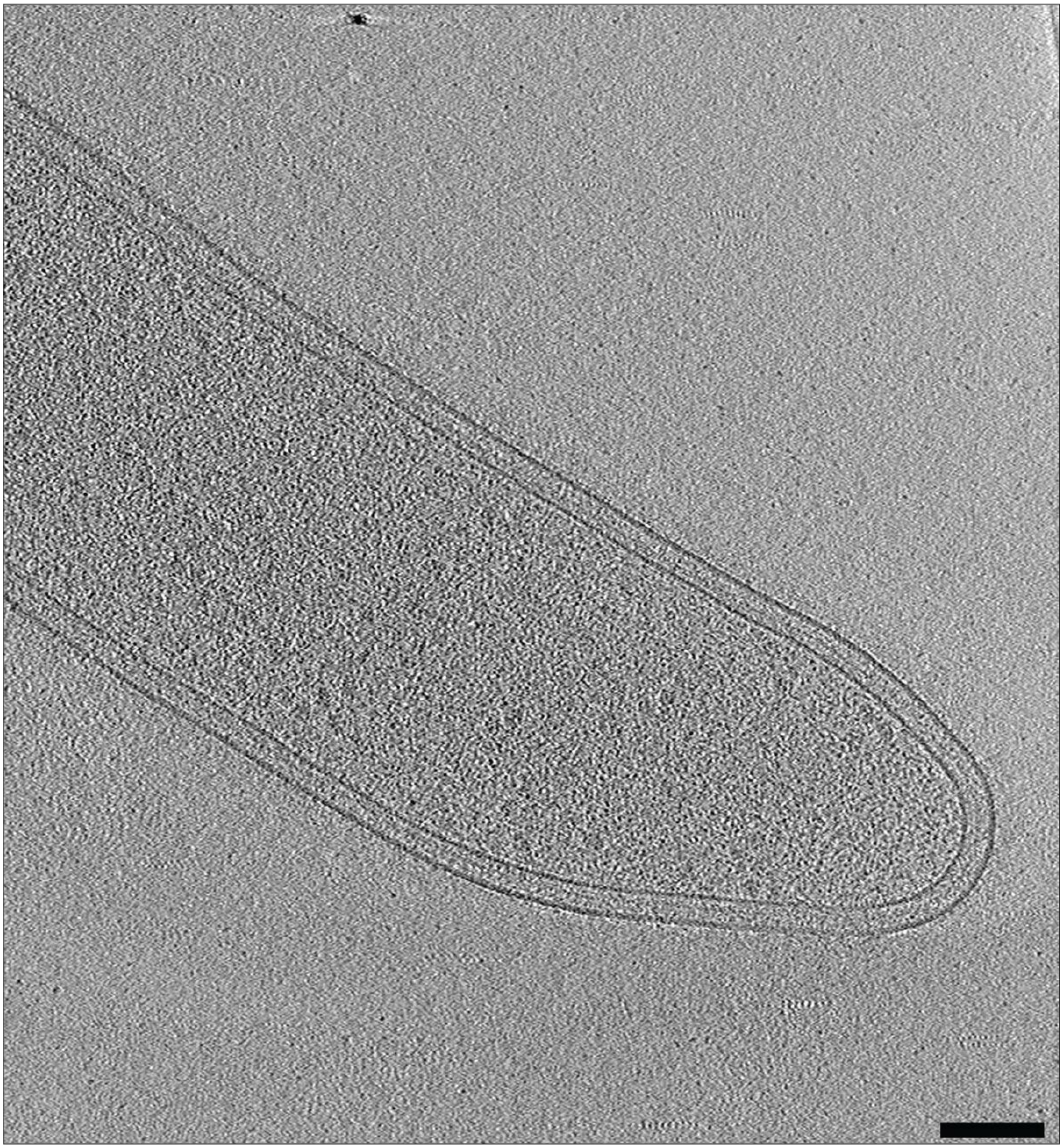

Coxiella burnetii

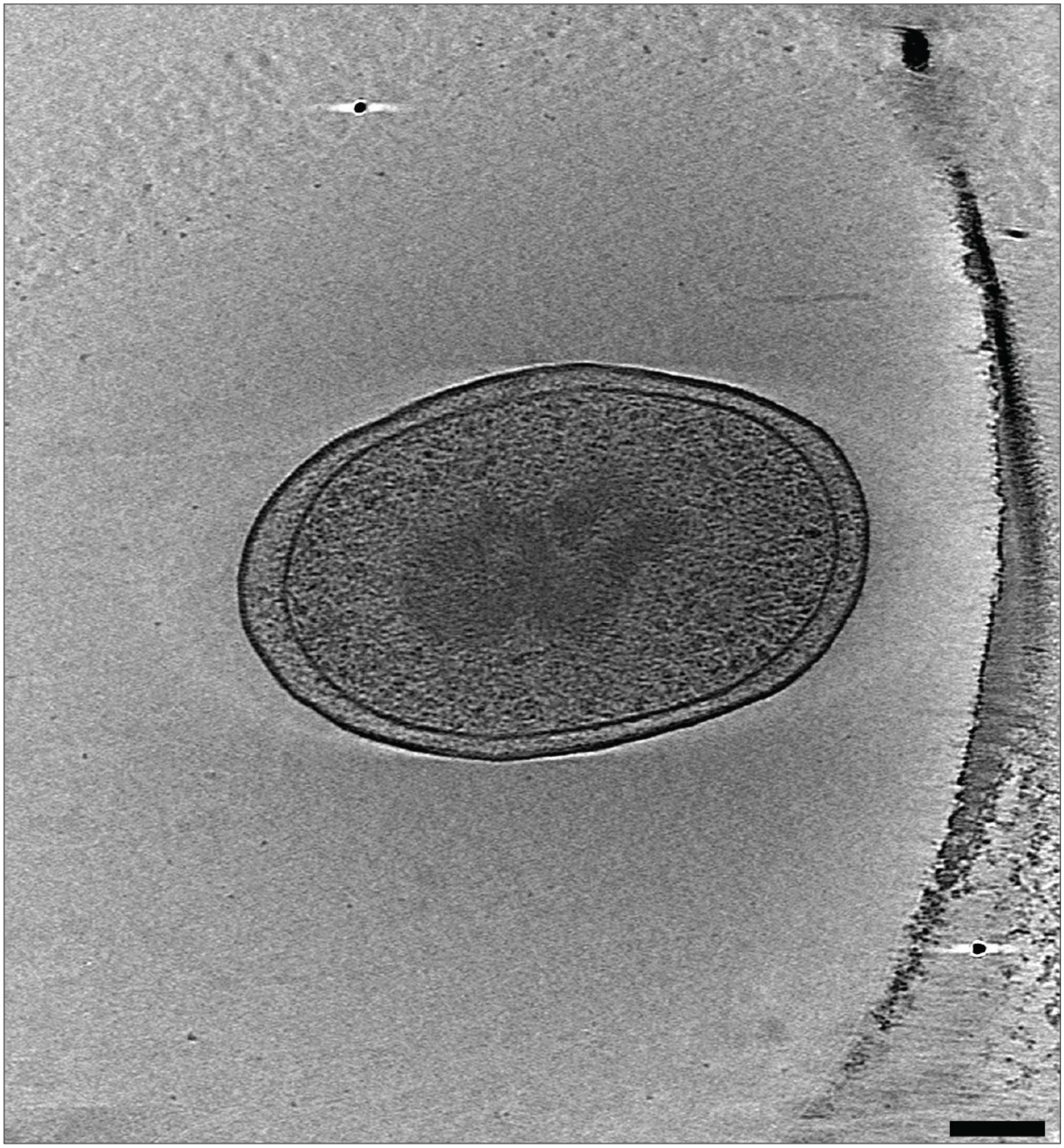

Amoebophilus asiaticus

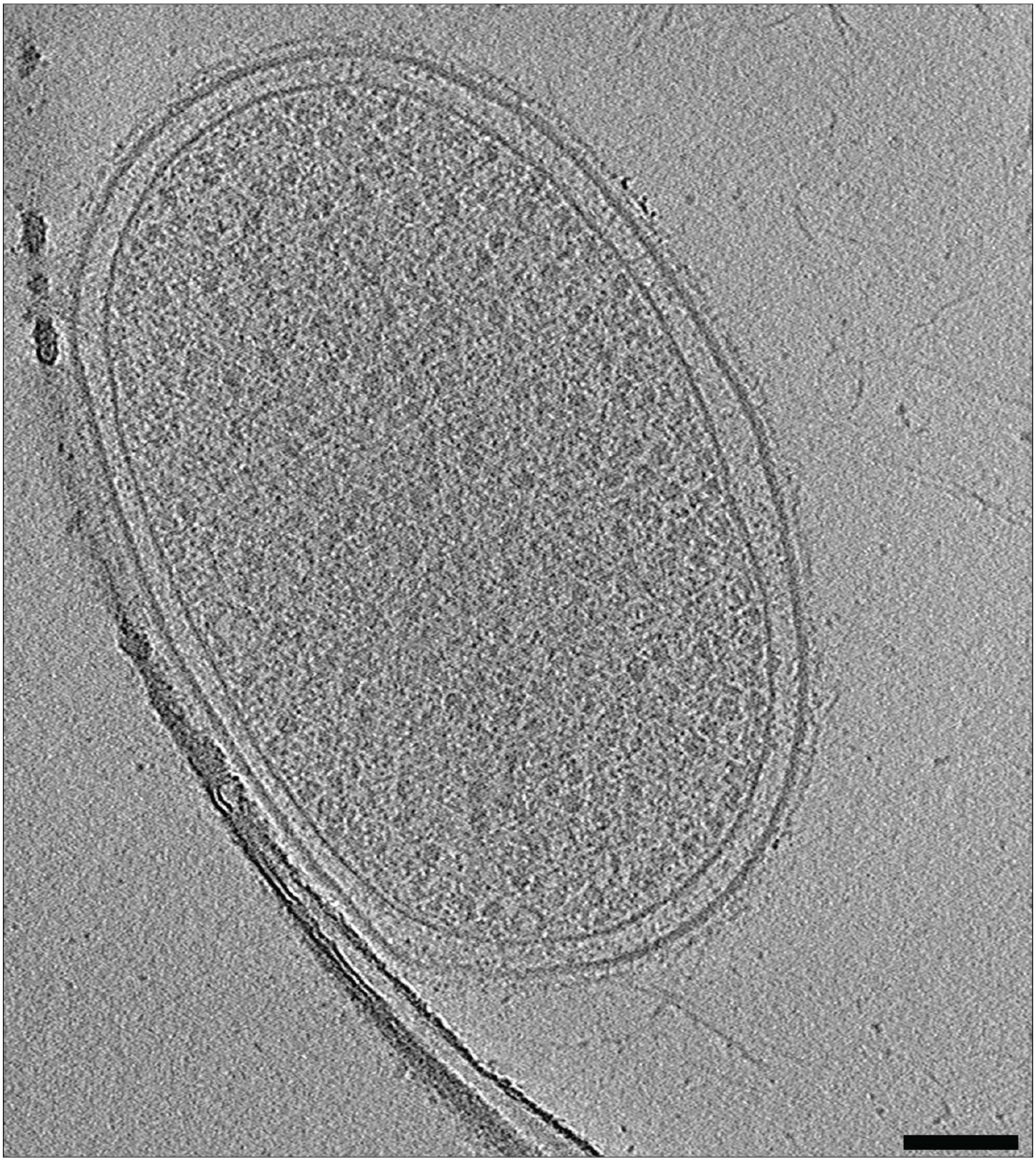

Figure S3: Species where no hat-like structures were identified. Slices through cryo-electron tomograms of species in which we found no hat-like structures. Note that the EPEC cell (strain

8612) is from a strain which has the NF-T3SScc but lacks the fT3SScc genes (see Materials and Methods). Scale bar is 100 nm.

Movie S1:

An electron cryo-tomogram of a partially lysed *E. coli* cell highlighting the presence of multiple hat-like complexes in the inner membrane (indicated by red circles).
